# A Predictive Nomogram for Early Death in Pheochromocytoma and Paraganglioma

**DOI:** 10.3389/fonc.2022.770958

**Published:** 2022-02-25

**Authors:** Huiyang Li, Kirellos Said Abbas, Basel Abdelazeem, Yao Xu, Yile Lin, Haixiao Wu, Vladimir P. Chekhonin, Karl Peltzer, Chao Zhang

**Affiliations:** ^1^ Department of Obstetrics & Gynecology, Tianjin Medical University General Hospital, Tianjin, China; ^2^ Tianjin Key Laboratory of Female Reproductive Health and Eugenics, Tianjin, China; ^3^ The Sino-Russian Joint Research Center for Bone Metastasis in Malignant Tumor, Tianjin, China; ^4^ Faculty of Medicine, Alexandria University, Alexandria, Egypt; ^5^ McLaren Health Care, Flint/Michigan State University, Michigan City, MI, United States; ^6^ Department of Bone and Soft Tissue Tumors, Tianjin Medical University Cancer Institute and Hospital, National Clinical Research Center for Cancer, Key Laboratory of Cancer Prevention and Therapy, Tianjin’s Clinical Research Center for Cancer, Tianjin, China; ^7^ Department of Basic and Applied Neurobiology, Federal Medical Research Center for Psychiatry and Narcology, Moscow, Russia; ^8^ Department of Psychology, University of the Free State, Turfloop, South Africa

**Keywords:** pheochromocytoma, paraganglioma, SEER (surveillance epidemiology and end results) database, nomogram, early death, survival analysis

## Abstract

**Background:**

Pheochromocytoma (PHEO) and paraganglioma (PGL) are relatively rare neuroendocrine tumors. The factors affecting patients with early death remain poorly defined. We aimed to study the demographic and clinicopathologic pattern and to develop and validate a prediction model for PHEO/PGL patients with early death.

**Methods:**

Data of 800 participants were collected from the Surveillance Epidemiology and End Results (SEER) database as a construction cohort, while data of 340 participants were selected as a validation cohort. Risk factors considered included the year of diagnosis, age at diagnosis, gender, marital status, race, insurance status, tumor type, primary location, laterality, the presence of distant metastasis. Univariate and multivariate logistic regressions were performed to determine the risk factors. R software was used to generate the nomogram. Calibration ability, discrimination ability, and decision curve analysis were analyzed in both construction and validation cohorts.

**Results:**

PHEO and PGL patients accounted for 54.3% (N=434) and 45.7% (N=366), respectively. More than half of tumors (N=401, 50.1%) occurred in the adrenal gland, while 16.9% (N=135) were in aortic/carotid bodies. For the entire cohort, the median overall survival (OS) was 116.0 (95% CI: 101.5-130.5) months. The multivariate analysis revealed that older age (*versus age younger than 31;* age between 31 and 60: OR=2.03, 95% CI: 1.03-4.03, *P=0.042*; age older than 60: OR=5.46, 95% CI: 2.68-11.12, *P<0.001*), female gender (*versus male gender;* OR=0.59, 95% CI: 0.41-0.87, *P=0.007*), tumor located in aortic/carotid bodies (*versus tumor located in adrenal gland;* OR=0.49, 95% CI: 0.27-0.87, *P=0.015*) and the presence of distant metastasis (*versus without distant metastasis;* OR=4.80, 95% CI: 3.18-7.23, *P<0.001*) were independent risk factors of early death. The predictive nomogram included variables: age at diagnosis, gender, primary tumor location, and distant metastasis. The model had satisfactory discrimination and calibration performance: Harrell’s C statistics of the prediction model were 0.733 in the construction cohort and 0.716 in the validation cohort. The calibration analysis showed acceptable coherence between predicted probabilities and observed probabilities.

**Conclusions:**

We developed and validated a predictive nomogram utilizing data from the SEER database with satisfactory discrimination and calibration capability which can be used for early death prediction for PHEO/PGL patients.

## Introduction

Pheochromocytoma (PHEO) and paraganglioma (PGL) are catecholamine-secreting neuroendocrine tumors abbreviated collectively in the literature as PHEO/PGL or PPGL (1–3). The incidence of PHEO/PGL is 2-8:1,000,000, and the prevalence is up to 1:2500 and a peak of incidence between the third and fifth decades (4–6). PHEO/PGL arises from chromaffin cells, but PHEO arises from the adrenal medulla and PGLs from the ganglia of the autonomic nervous system (1, 3, 7). 40% of PPGL have hereditary germline mutation, and 60% are sporadic. 20% -75% of hereditary cases and 8% of sporadic cases diagnosed as metastasis at presentation. Metastasis occurs in 15 -17% of PPGLs cases, 2-25% in PHEOs, and 2.4-60% in PGL (6, 8–10). PGLs can be found in any site in the abdomen arising from sympathetic or parasympathetic ganglia; 70-80% of PGLs arise from the Zuckerkandl organ near the aortic bifurcation, 20% from head and neck, and 10% in the mediastinal and pericardial region with less frequent in the retroperitoneal area (7, 11). Incidental discovery of PHEO/PGL represents 10-49% of the cases through imaging techniques for other reasons (6).

Survival of malignant tumors may vary depending on the site of the primary tumor and the site of metastasis. The survival rate of metastatic PHEO/PGL varies widely from 12-84% (12–14). Moskovic et al. reviewed the record of 19 patients with malignant paragangliomas originated in head & neck in one single institution from 1970 to 2005. They reported that the 5-year survival rate was 84%, and the 10-year survival rate was 53% (15). Sethi et al. used National Cancer Institute Surveillance Epidemiology and End Results (SEER) database from 1973 to 2009 to conduct a retrospective cohort study for 86 patients with malignant head & neck paragangliomas. They concluded that age and tumor stage are important predictors of survival. The overall 5-year survival rate was 65%, the survival among patients with regional metastases (n = 47) was 82%, compared to those with distant metastases (n = 39) was 41% (16). Another study using the SEER database from 1988-2009 by Gofferdo et al. reported that PHEO had 54.0% overall survival and 73.5% disease-specific survival *P* < 0.001 compared to 73.3% overall survival and 80.5% disease-specific survival *P* = 0.118 for PGL (17). de Flines et al. estimated the mean survival to be 26.4 years after diagnosis in a cohort of 86 patients with PGL in head & neck (18). These studies provided us with information on overall survival. However, to our knowledge, no studies have thoroughly discussed risk factors of early death.

In this study, we analyzed data of 1,140 patients from the SEER database to evaluate the overall survival and early death incidence in patients with PHEO/PGL. In addition, logistic regression analysis was used to identify risk factors and construct nomograms of PHEO/PGL early death.

## Patients And Methods

### Data Source and Cohort Selection

The data were extracted from the SEER database, in which American population covers approximately 28%. The SEER program provides information on cancer statistics to reduce the cancer burden, and all authors were permitted to access the original data without informed consent. The present study was conducted in accordance with the most recent revision of the Helsinki Declaration or comparable ethical standards.

The database, which was named *Incidence - SEER 18 Regs Research Data + Hurricane Katrina Impacted Louisiana Cases, Nov 2018 Sub (1975-2016 varying)*, was selected as the data source for the present study. Patients diagnosed with malignant PHEO and PGL between 1975 and 2016 were extracted from the database according to the variable of *Site* and *Morphology. ICD-O-3 His/behav, malignant* (PHEO: 8700/3: Pheochromocytoma, malignant. PGL: 8680/3: Paraganglioma, malignant; 8683/3: Gangliocytic paraganglioma, malignant; 8693/3: Extra-adrenal paraganglioma, malignant). Patients without detailed information about survival time were excluded from the study.

### Demographic and Clinicopathologic Variables

Demographic and clinicopathologic variables included in the present study were as follows: year of diagnosis (1975-2003, 2004-2009 and 2010-2016), age at diagnosis (0-30, 31-60 and over 60), gender (male and female), marital status (married, unmarried and unknown), race (white, black, others and unknown), insurance status (insured, uninsured and unknown), tumor type (PHEO and PGL), primary location (adrenal gland, aortic/carotid bodies, and other sites), laterality (unilateral and bilateral), the presence of distant metastasis (no and yes).

### Statistical Analyses

In the present study, the total cohort was randomly divided into the construction and validation cohorts (ratio 7:3). Early death was defined as overall survival time ≤ 36 months after initial diagnosis. The construction cohort was used to identify the risk factors of early death in PPGL patients and construct a nomogram. The performance of the model was evaluated in the validation cohort. Quantitative data were described as mean ± standard deviation (SD) while categorical variables were presented as number and percentage (N, %). The univariate and multivariate logistic regression model was performed to determine the risk factors. R software was used to formulate the nomogram. Harrell’s concordance index (C-index) and receiver operating characteristic (ROC) was calculated to evaluate the performance of the model and C-index or Area Under Curve (AUC) larger than 0.700 indicated satisfactory performance of discrimination. The calibration curves were performed to evaluate the calibration ability of the nomogram.

SEER*Stat software (www.seer.cancer.gov/seerstat) version 8.3.9 was used to generate data, and the IBM SPSS Statistics (version 26.0, Armonk, NY, USA) was used for statistical analyses. The construction of the prognostic nomogram and subsequent validation were performed with R version 4.0.0 (R Foundation for Statistical Computing, Vienna, Austria; www.r-project.org). All statistical tests were two-sided, and P<0.05 was considered significant.

## Results

### Demographic and Clinicopathologic Characteristics

A total of 1,140 PHEO/PGL qualified patients were identified in SEER database and selected in the present study (800 and 340 in construction cohort and validation cohort, respectively). As for patients in the construction cohort, the mean age was 50.1 ± 17.6 years, with a slight predominance for male (N=411, 51.4%) and married (N=423, 52.9%) patients. The majority of the construction population were white race (N=594, 74.3%) or black race (N=130, 16.3%). Since insurance status information was not available before 2007, more than half of cases were in unknown status (N=451, 56.4%). PHEO and PGL patients accounted for 54.3% (N=434) and 45.7% (N=366), respectively. More than half of tumors (N=401, 50.1%) occurred in the adrenal gland, while 16.9% (N=135) were located in aortic/carotid bodies. In addition, 264 (33.0%) cases were diagnosed with tumors located in other sites. Almost all of the tumors were unilateral (N=755, 94.4%). The percentage of patients with distant metastasis was 20.9% (N=167). Demographic and clinicopathologic details were shown in **Table 1**.

**Table 1:** Demographic and clinicopathologic characteristics in the present study.

Subject Characteristics	Total Cohort	Construction Cohort	Validation Cohort	χ^2^	*P*
	N (%)	N (%)	N (%)		
**Age (years, mean = 61.5 ± 13.7; median = 61)**					
≤30	173 (15.2)	119 (14.9)	54 (15.9)	0.198	*0.906*
31-60	630 (55.3)	443 (55.4)	187 (55.0)		
>60	337 (29.6)	238 (29.8)	99 (29.1)		
**Year of diagnosis**					
1975-2003	457 (40.1)	327 (40.9)	130 (38.2)	3.161	*0.206*
2004-2009	324 (28.4)	215 (26.9)	109 (32.1)		
2010-2016	359 (31.5)	258 (32.3)	101 (29.7)		
**Gender**					
Male	598 (52.5)	411 (51.4)	187 (55.0)	1.257	*0.262*
Female	542 (47.5)	389 (48.6)	153 (45.0)		
**Marital status**					
Married	601 (52.7)	423 (52.9)	178 (52.4)	0.450	*0.798*
Unmarried	468 (41.1)	325 (40.6)	143 (42.1)		
Unknown	71 (6.2)	52 (6.5)	19 (5.6)		
**Race**					
White	833 (73.1)	594 (74.3)	239 (70.3)	2.670	*0.445*
Black	198 (17.4)	130 (16.3)	68 (20.0)		
Others	98 (8.6)	69 (8.6)	29 (8.5)		
Unknown	11 (1.0)	7 (0.9)	4 (1.2)		
**Insurance**					
Insured	474 (41.6)	331 (41.4)	143 (42.1)	0.064	*0.968*
Uninsured	26 (2.3)	18 (2.3)	8 (2.4)		
Unknown	640 (56.1)	451 (56.4)	189 (55.6)		
**Tumor type**					
PHEO	613 (53.8)	434 (54.3)	179 (52.6)	0.247	*0.619*
PGL	527 (46.2)	366 (45.7)	161 (47.4)		
**Primary location**					
Adrenal gland	577 (50.6)	401 (50.1)	176 (51.8)	0.489	*0.783*
Aortic/carotid bodies	194 (17.0)	135 (16.9)	59 (17.4)		
Other sites	369 (32.4)	264 (33.0)	105 (30.9)		
**Laterality**					
Unilateral	1078 (94.6)	755 (94.4)	323 (95.0)	0.181	*0.670*
Bilateral	62 (5.4)	45 (5.6)	17 (5.0)		
**Distant metastasis**					
No	910 (79.8)	633 (79.1)	277 (81.5)	0.815	*0.367*
Yes	230 (20.2)	167 (20.9)	63 (18.5)		
**Early death**					
No	877 (76.9)	611 (76.4)	266 (78.2)	0.465	*0.495*
Yes	263 (23.1)	189 (23.6)	74 (21.8)		

PHEO, pheochromocytoma; PGL, paraganglioma.

### Survival of Patients and Risk Factors of Early Death

For the entire 1,140 patients, the median overall survival (OS) was 116.0 (95% CI: 101.5-130.5) months. The 1-year, 3-year, 5-year and 10-year OS was 87.4%, 75.3%, 66.6% and 48.9%, respectively. As for construction cohort, the median OS was 116.0 (95% CI: 99.6-132.4) months. The 1-year, 3-year, 5-year and 10-year OS was 87.0%, 74.6%, 65.4% and 49.1%, respectively.

Parameters associated with the development of early death included age at diagnosis, year of diagnosis, gender, marital status, insurance status, primary tumor location, distant metastasis. The multivariate analysis identified that older age (*versus age younger than 31;* age between 31 and 60: OR=2.03, 95% CI: 1.03-4.03, *P=0.042*; age older than 60: OR=5.46, 95% CI: 2.68-11.12, *P<0.001*), year of diagnosis (*versus year between 1975-2003;* year between 2010-2016: OR=0.42, 95% CI: 0.20-0.89, *P=0.024*), female gender (*versus male gender;* OR=0.59, 95% CI: 0.41-0.87, *P=0.007*), tumor located in aortic/carotid bodies (*versus tumor located in adrenal gland;* OR=0.49, 95% CI: 0.27-0.87, *P=0.015*) and the presence of distant metastasis (*versus without distant metastasis;* OR=4.80, 95% CI: 3.18-7.23, *P<0.001*) were independent risk factors of early death. More details about the logistic regression analysis were shown in **Table 2**.

**Table 2:** Logistic regression model for analyzing the risk factors for early death in patients diagnosed with pheochromocytoma and paraganglioma.

Subject Characteristics	Univariate		Multivariate	
	OR (95% CI)	*P-value*	OR (95% CI)	*P-value*
**Age**				
≤30	1 (reference)	*1.00*	1 (reference)	*1.00*
31-60	2.14 (1.15-3.97)	*0.016*	2.03 (1.03-4.03)	*0.042*
>60	4.45 (2.36-8.39)	*<0.001*	5.46 (2.68-11.12)	*<0.001*
**Year of diagnosis**				
1975-2003	1 (reference)	*1.00*	1 (reference)	*1.00*
2004-2009	0.54 (0.36-0.82)	*0.004*	0.74 (0.42-1.33)	*0.314*
2010-2016	0.49 (0.33-0.72)	*<0.001*	0.42 (0.20-0.89)	*0.024*
**Gender**				
Male	1 (reference)	*1.00*	1 (reference)	*1.00*
Female	0.66 (0.47-0.92)	*0.013*	0.59 (0.41-0.87)	*0.007*
**Marital status**				
Married	1 (reference)	*1.00*	1 (reference)	*1.00*
Unmarried	0.65 (0.46-0.92)	*0.014*	0.96 (0.64-1.44)	*0.835*
Unknown	0.21 (0.08-0.60)	*0.004*	0.42 (0.14-1.23)	*0.113*
**Race**				
White	1 (reference)	*1.00*	1 (reference)	
Black	0.83 (0.53-1.33)	*0.444*	–	–
Others	1.12 (0.64-1.99)	*0.689*	–	–
Unknown	1.27 (0.24-6.63)	*0.774*	–	–
**Insurance**				
Insured	1 (reference)	*1.00*	1 (reference)	*1.00*
Uninsured	0.51 (0.12-2.28)	*0.379*	0.87 (0.18-4.10)	*0.858*
Unknown	1.52 (1.08-2.14)	*0.017*	0.91 (0.48-1.74)	*0.781*
**Tumor type**				
PHEO	1 (reference)	*1.00*	1 (reference)	*1.00*
PGL	0.77 (0.55-1.07)	*0.114*	–	–
**Primary location**				
Adrenal gland	1 (reference)	*1.00*	1 (reference)	*1.00*
Aortic/carotid bodies	0.48 (0.28-0.82)	*0.007*	0.49 (0.27-0.87)	*0.015*
Other sites	1.02 (0.71-1.45)	*0.926*	0.88 (0.60-1.31)	*0.543*
**Laterality**				
Unilateral	1 (reference)	*1.00*	1 (reference)	*1.00*
Bilateral	1.34 (0.69-2.60)	*0.394*	–	–
**Distant metastasis**				
No	1 (reference)	*1.00*	1 (reference)	*1.00*
Yes	4.58 (3.17-6.61)	*<0.001*	4.80 (3.18-7.23)	*<0.001*

PHEO, pheochromocytoma; PGL, paraganglioma.

### Construction and Validation of the nomogram

**Figure 1:**
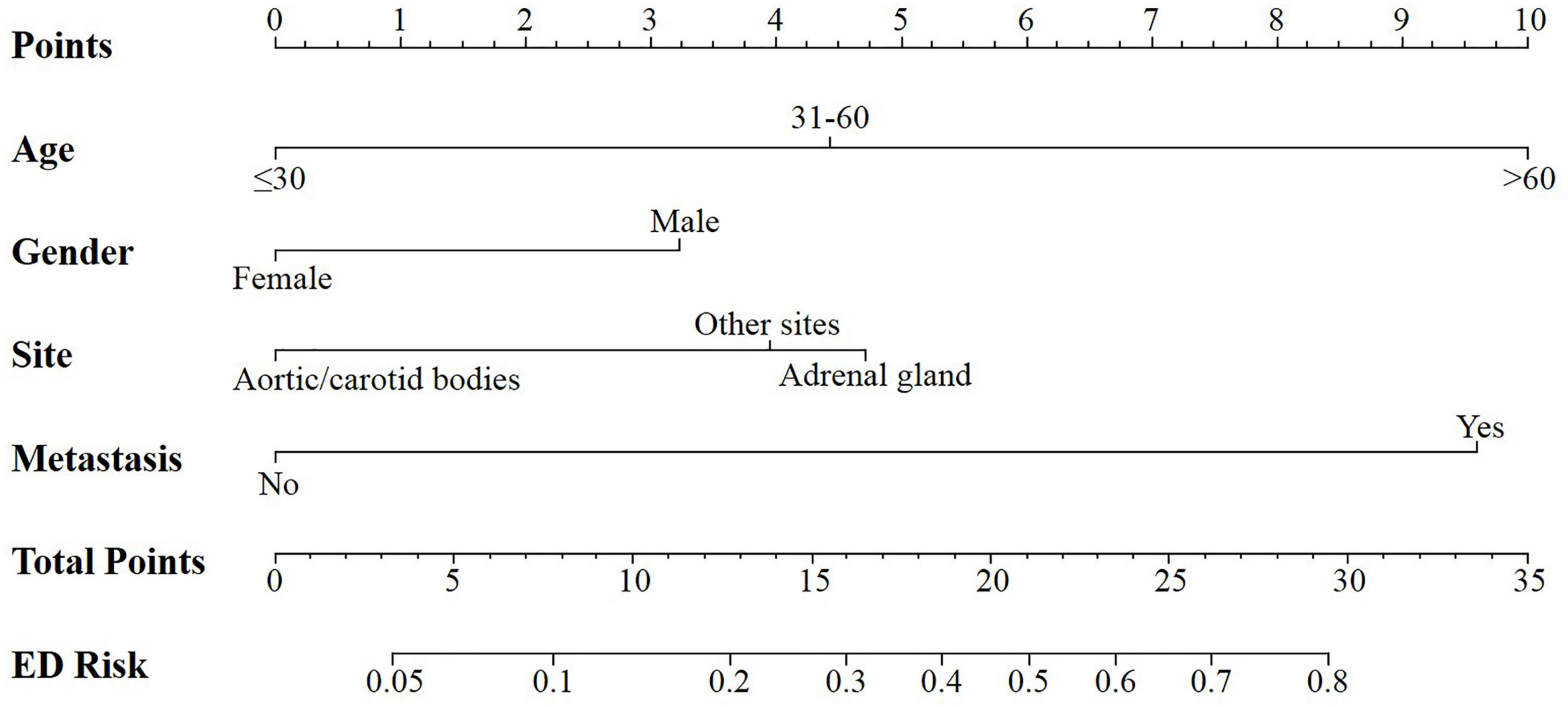
Nomogram for predicting all cause early death in patients with pheochromocytoma and paraganglioma.

**Figure 2:**
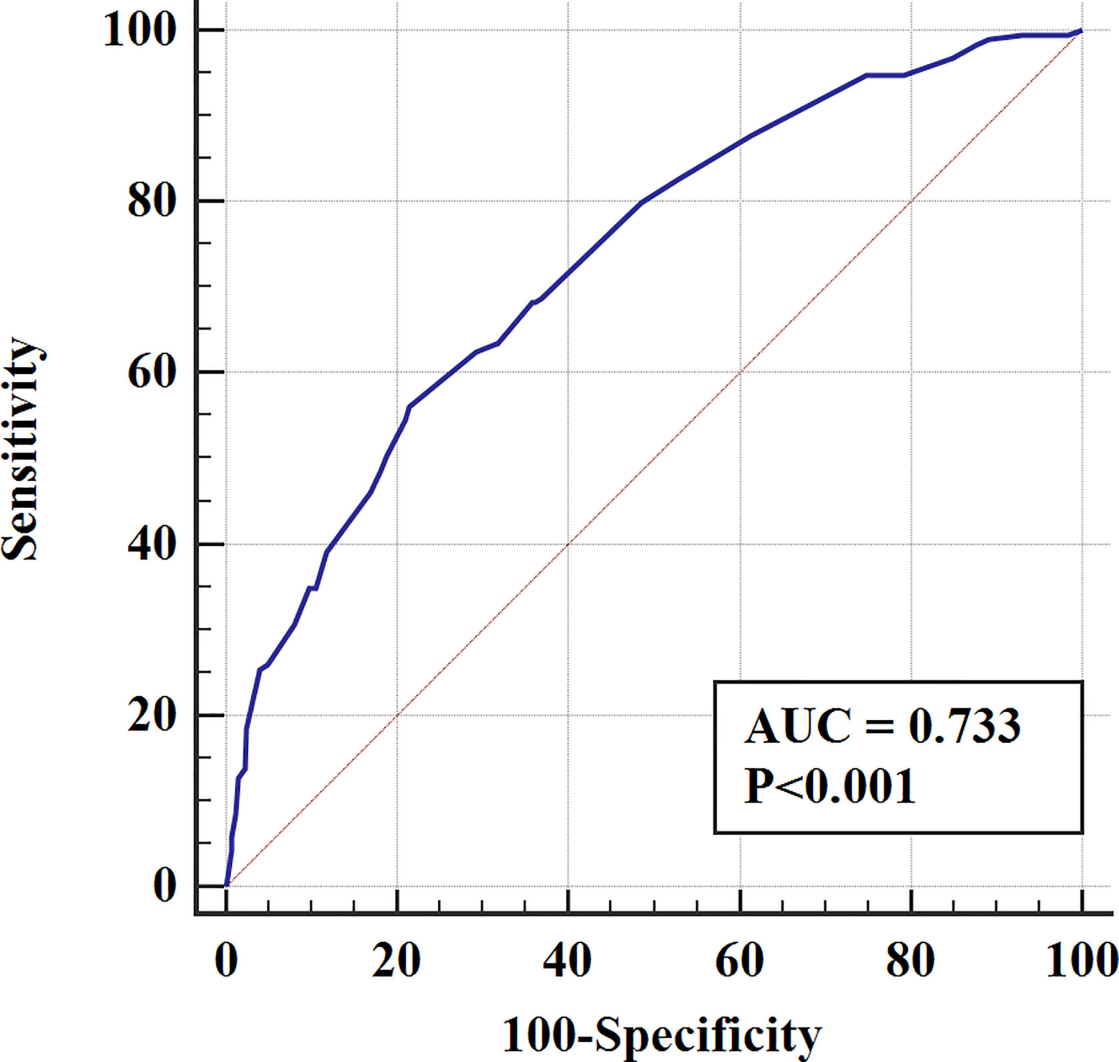
The ROC curve for assessing the discrimination of the nomogram in predicting all cause of early death in the construction cohort.

**Figure 3:**
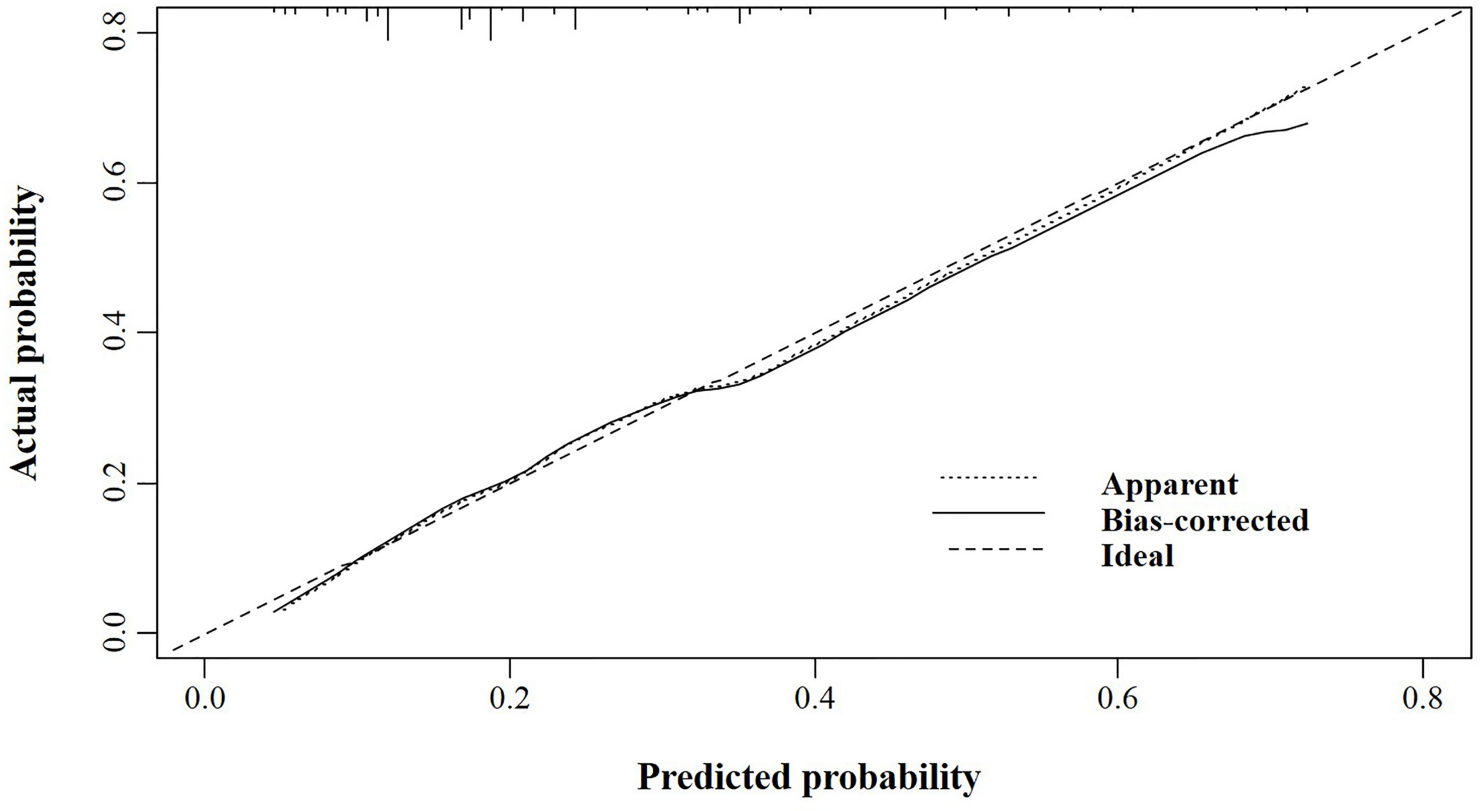
The calibration curve for assessing the calibration of the nomogram in predicting all cause of early death in the construction cohort.

According to the logistic regression model findings, variables including age at diagnosis, gender, primary tumor location, and distant metastasis, were selected to construct the predictive nomogram (**Figure 1**). Considering the clinical applicability of the nomogram, the variable of the diagnostic year was not incorporated into the model. The C-index for the construction cohort was 0.733 (95%CI: 0.701-0.764), and the ROC curve was shown in **Figure 2**. The calibration curve revealed proper agreement between the predicted and observed probabilities with the calibration curve close to the 45-degree line (**Figure 3**).

**Figure 4:**
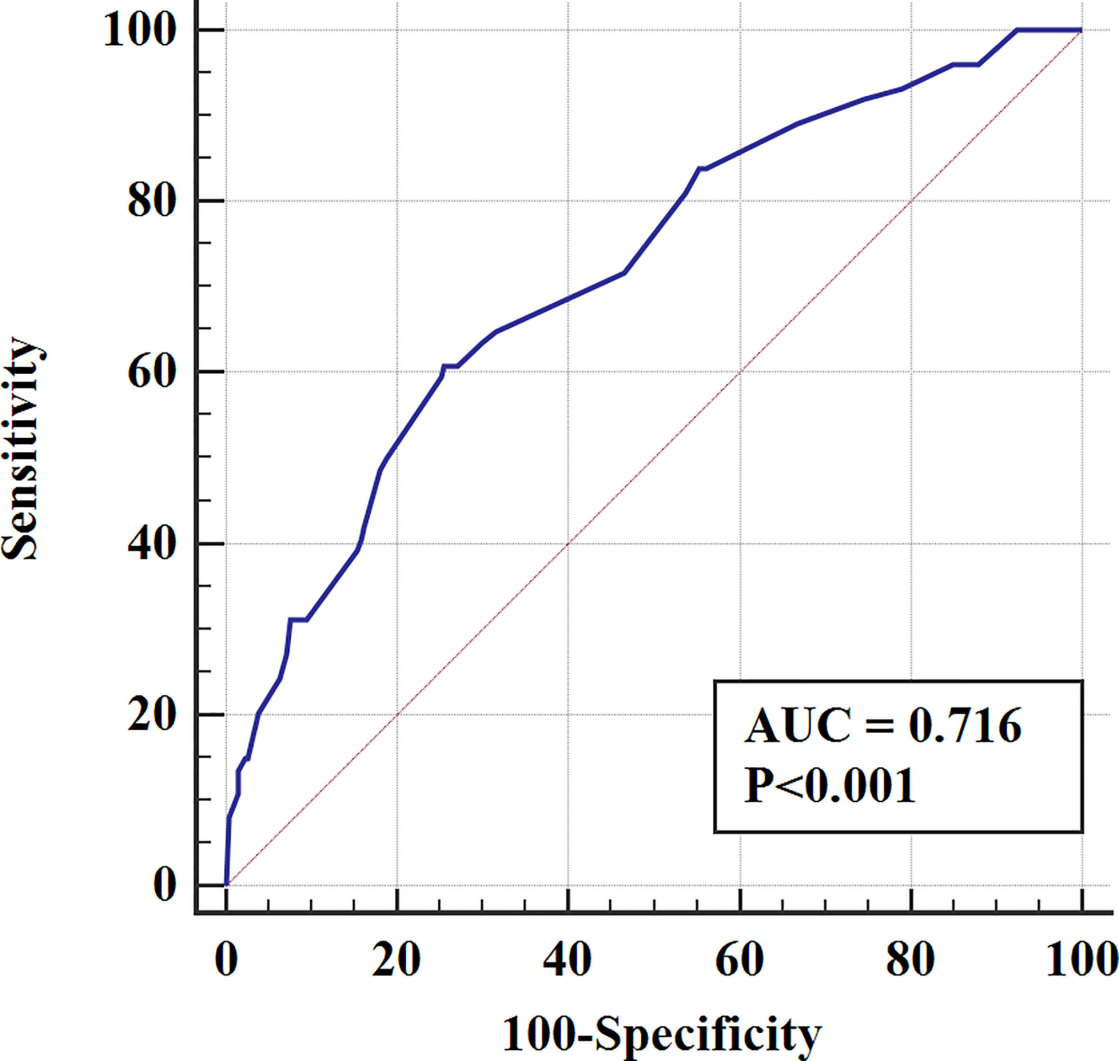
The ROC curve for assessing the discrimination of the nomogram in predicting all cause of early death in the validation cohort.

**Figure 5:**
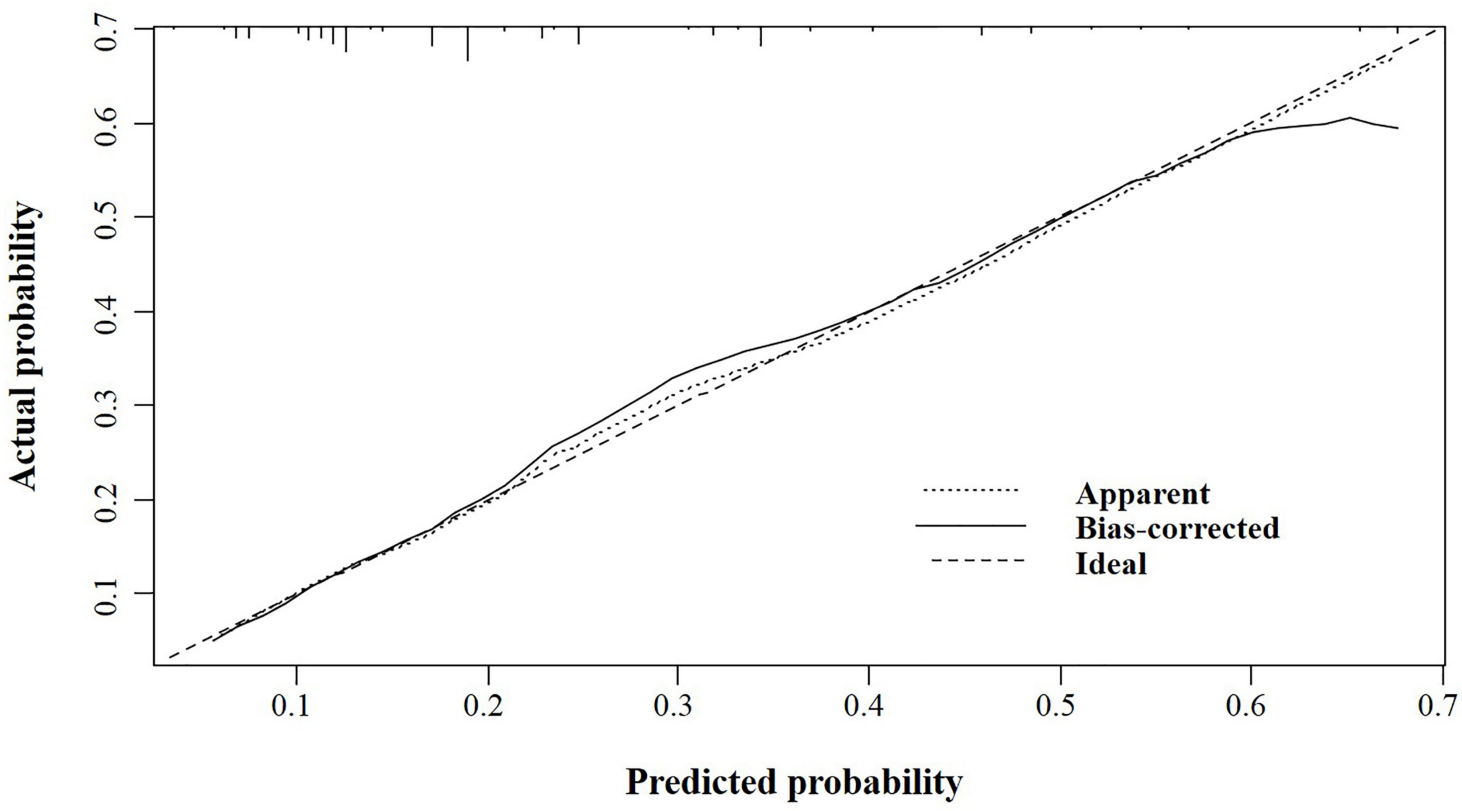
The calibration curve for assessing the calibration of the nomogram in predicting all cause of early death in the validation cohort.

In the validation cohort, the nomogram showed satisfactory strength of discrimination. The C-index was 0.716 (95%CI: 0.665-0.763), and the ROC curve was shown in **Figure 4**. Excellent calibration ability was achieved with a calibration curve close to the 45-degree line (**Figure 5**).

## Discussion

Investigating early death incidence and the associated risk factor is vital for improving survival in patients with PHEO/PGL. To the best of our knowledge, this cohort study represents the largest cohort regarding the number of patients, with a total of 1140 patients, and it is the first study to evaluate the risk factor associated with early death using the SEER database.

The current study found several factors associated with early death in patients with PHEO/PGL, including age at diagnosis, year of diagnosis, gender, marital status, insurance status, primary tumor location, and distant metastasis. The age older than 60 years and the presence of distant metastasis were associated with a higher odds ratio for early death. In contrast, female gender, recent years of diagnosis, and location in aortic/carotid bodies are associated with a lower odds ratio for early death.

In the present study, we found that age older than 60 years and presence of distant metastasis was independent risk factors of early death, respectively. De Filpo et al. conducted a retrospective study of 20 patients and found that older age is associated with poor prognosis as overall survival from the initial diagnosis (*P* = 0.028) and overall survival from the diagnosis of metastases (*P* < 0.001) was lower in older patients (19). Another study by Mei et al. reported that the overall survival was 63 months in 277 PHEO/PGL patients with age more than 60 years, using both SEER and Cancer Genome Atlas (TCGA) database (20). Contrary to the risk mentioned above factors, female gender (OR=0.59) and the presence of the primary location at the aortic/carotid bodies (OR=0.49) were also independent risk factors but was associated with lower odds of early death (OR=0.59, 95% CI: 0.41-0.87, *P*=0.007), (OR=0.49, 95% CI: 0.27-0.87, *P*=0.015), respectively. The underlying reasons should be investigated in future studies. Other factors to be considered are the year of diagnosis and socioeconomic status. Patients diagnosed in the current era have more access to health care besides the advancement in the health system worldwide and patient care. In addition, the patients who are married and insured will have more support mentally and financially (21, 22).

Nomograms are effective tools in quantifying risk factors with subsequent risk stratification and prediction in multiple cancers (21, 23–26). They are supposed to provide clinicians with a feasible screening tool to improve clinical outcomes, decision-making, and treatment regimens for PPGL patients. Our nomogram has satisfactory discriminant power compared to previous literature.

The present study has some limitations. Since data on recurrence, different treatment modalities, and hormonal status were not available in the SEER database, we were not able to calculate disease-free survival analysis and survival of hormonal subtypes. Furthermore, no information was provided on medical complications and co-morbidities that are important for assessing the quality of life, mental health, and illness burden. Therefore, our preliminary results and predictive models should be validated externally.

## Conclusion

We used the SEER database to conduct a retrospective study to evaluate the overall survival and the risk factor affecting early death in patients with PHEO/PGL. We included 1,140 patients and found that the median overall survival was 116 months. Our data showed that age older than 60 years and presence of distant metastasis were independent risk factors and associated with higher odds of early death; meanwhile, female gender, recent years of diagnosis, and location in aortic/carotid bodies were also independent risk factors but were associated with a lower odds ratio for early death. The established nomogram can help oncologists in screening patients who are at higher risk for tailoring treatment plans.

## Data Availability Statement

The original contributions presented in the study are included in the article/supplementary material. Further inquiries can be directed to the corresponding author.

## Author Contributions

HL and CZ conceptualized and designed the study. KA and BA provided analyzed and interpreted the data. YX, HW, and YL provided statistical support, including data collection and assembly. VC and KP reviewed the framework and content of the discussion. All authors contributed to the article and approved the submitted version.

## Funding

The present study was sponsored by Natural Science Foundation of China (82011530050).

## Conflict of Interest

The authors declare that the research was conducted in the absence of any commercial or financial relationships that could be construed as a potential conflict of interest.

## Publisher’s Note

All claims expressed in this article are solely those of the authors and do not necessarily represent those of their affiliated organizations, or those of the publisher, the editors and the reviewers. Any product that may be evaluated in this article, or claim that may be made by its manufacturer, is not guaranteed or endorsed by the publisher.
